# *Burkholderia pseudomallei* Isolates in 2 Pet Iguanas, California, USA

**DOI:** 10.3201/eid2002.131314

**Published:** 2014-02

**Authors:** Ashley M. Zehnder, Michelle G. Hawkins, Marilyn A. Koski, Barry Lifland, Barbara A. Byrne, Alexandra A. Swanson, Michael P. Rood, Jay E. Gee, Mindy Glass Elrod, Cari A. Beesley, David D. Blaney, Jean Ventura, Alex R. Hoffmaster, Emily S. Beeler

**Affiliations:** School of Veterinary Medicine, University of California, Davis, Davis, California, USA (A.M. Zehnder, M.G. Hawkins, M.A. Koski, B.A. Byrne);; Stanford University School of Medicine, Stanford, California, USA (B. Lifland);; Los Angeles County Department of Public Health, Los Angeles, California, USA (A.A. Swanson, M.P. Rood, E.S. Beeler);; Centers for Disease Control and Prevention, Atlanta, Georgia, USA (J.E. Gee, M.G. Elrod, C.A. Beesley, D.D. Blaney, A.R. Hoffmaster);; Sacramento County Department of Public Health, Sacramento, California, USA (J. Ventura)

**Keywords:** Burkholderia pseudomallei, iguana, zoonoses, abscess, melioidosis, bacteria

## Abstract

*Burkholderia pseudomallei*, the causative agent of melioidosis*,* was isolated from abscesses of 2 pet green iguanas in California, USA. The international trade in iguanas may contribute to importation of this pathogen into countries where it is not endemic and put persons exposed to these animals at risk for infection.

*Burkholderia pseudomallei*, a gram-negative bacterium, is the causative agent of melioidosis. Melioidosis is endemic in countries in Southeast Asia and in northern Australia, and has been sporadically reported from Central and South America (*1*). In the United States, most case-patients have traveled to disease-endemic areas (*2*).

*B. pseudomallei* infection occurs through direct cutaneous inoculation with soil or water containing *B. pseudomallei* and through ingestion or inhalation of aerosolized bacteria. In humans, the incubation period is typically 1–21 days, but some patients demonstrate clinical signs years after exposure (*1*). Acute melioidosis can manifest as a severe pneumonia and septicemia, with death rates >40% in countries where access to medical care is limited. In chronic melioidosis, abscesses occur in various organs, including the lungs, liver, spleen, and cutaneous sites (*1*,*3*). In animals, abscesses and acute illness are common (*4*).

*B. pseudomallei* is classified by US federal agencies as a tier 1 select agent. Tier 1 agents are believed to pose the greatest threat for deliberate misuse and potential harm to public health. Multiple regulations restrict access to these agents and reduce the risk of their release from secure settings (*5*). Infection is generally diagnosed by culture. Commercially available bacterial identification systems may provide initial identification; however, *B. pseudomallei* may be misidentified by some systems (*6*). Other identification tests are available, including PCR and antigen detection; these are not commonly used outside disease-endemic regions (*3*,*7*,*8*).

##  Case Reports

A 2.5-year-old female green iguana (*Iguana iguana*) was referred to the William R. Pritchard Veterinary Medical Teaching Hospital, University of California, Davis, in 2007 for evaluation of a coelomic mass and leukocytosis (40.0–42.0 × 10^3^ cells/mL [reference 3–10 × 10^3^ cells/mL]). Radiographs and ultrasound results confirmed multiple hepatic masses. Initial aspiration and culture of 1 mass yielded 3 colonies of a *Bacillus* spp. Empirical antimicrobial drug therapy with ceftazidime (20 mg/kg intramuscularly every 48 h for 30 d) was initiated. The iguana was brought for treatment of additional coelomic masses 6 months later. Aspiration of the masses and culture of the sample yielded small colonies of suspected *B. pseudomallei*; blood samples collected for culture were negative for the organism. Results of in-house biochemical testing and PCR were consistent with *B. pseudomallei* or *B. mallei*. The culture was submitted to Stanford University School of Medicine, Department of Comparative Medicine. The Biolog Dangerous Pathogens Database ID system (http://info.biolog.com/VetCampaign_vet_alt.html) identified *B. pseudomallei*. Laboratory Response Network PCR protocols and biochemical testing performed at the Santa Clara Public Health Laboratory (Santa Clara, CA, USA) confirmed *B. pseudomallei*. 

The isolate was then sent to the Centers for Disease Control and Prevention (CDC) for further characterization by multilocus sequence typing (MLST), a method of molecular subtyping that compares sequences from 7 housekeeping genes (*9*). This isolate’s sequence type (ST) isolate is ST518 (http://bpseudomallei.mlst.net/). No environmental testing was performed. Treatment included trimethoprim/sulfamethoxazole (20 mg/kg by mouth every 24 h) and doxycycline (10 mg/kg by mouth every 24 h), according to published treatment regimens and in-house susceptibility testing (*7*,*10*–*12*). Against recommendations, therapy was discontinued by the owner after 3 months. Re-evaluation of the patient 13 months after diagnosis revealed persistent leukocytosis. Euthanasia of the iguana was recommended, but the owner declined. The iguana died at home ≈2.5 years after initial diagnosis; a necropsy was not permitted.

In December 2012, a 1.6-year-old female green iguana was brought to a veterinarian in Los Angeles County, California, ≈400 miles south of the first case. An abscess, ≈40 mm in diameter, appeared on the iguana’s left shoulder 2 days after it fell from a height of 1 meter. The abscess was surgically removed and submitted to a commercial veterinary laboratory, which cultured β-hemolytic *Streptococcus* spp. and large numbers of unidentified gram-negative rods. Marbofloxacin (7.5 mg/kg by mouth, every other day, for 40 days) was prescribed. The laboratory forwarded the unidentified isolate to the Sacramento Public Health Laboratory, which identified it as *B. pseudomallei* by using Laboratory Response Network PCR protocols and biochemical testing. The isolate was forwarded to CDC where MLST was performed. The isolate was ST518, which matched the isolate from the first infected iguana and another isolate recovered from a tourist from Arizona who had been infected in Costa Rica in 2010 (http://bpseudomallei.mlst.net/).

Staff from Los Angeles County Department of Public Health visited the iguana owner’s home 3 weeks after the iguana completed marbofloxacin treatment. The iguana had a firm swelling on the left shoulder that was ≈25-mm in diameter, with a central flat, red crust that was 5 mm in diameter (Figure). The owner had not yet disinfected the animal’s housing. Sterile rayon swabs in liquid Amies medium were used to collect swab specimens from the iguana: 1 from the bottom of the feet, 1 from inside the cloacal opening (vent), and 2 from the red crust on the shoulder swelling. Four specimens were also collected from the iguana’s housing: 50 mL of water from its aluminum water bowl, a fecal sample in the water, a cloth containing feces from its tank, and a biofilm sample, recovered with a sterile polyurethane foam swab, from the inside of the emptied water bowl. The 8 samples underwent culture and type III secretion system real-time PCR for *B. pseudomallei* (*13*). PCR results from the water sample, the water bowl biofilm, and a shoulder swab specimen were positive. *B. pseudomallei* was cultured from the second shoulder swab specimen. CDC confirmed the isolate as *B. pseudomallei*. The owner euthanized the iguana, and tissue samples were collected in a Biosafety Level 3 cabinet at Los Angeles County Department of Public Health. The iguana’s shoulder swelling had grown to ≈30 mm in diameter, and multiple 5-mm yellow masses were found in the iguana’s liver, lungs, and spleen.

**Figure:**
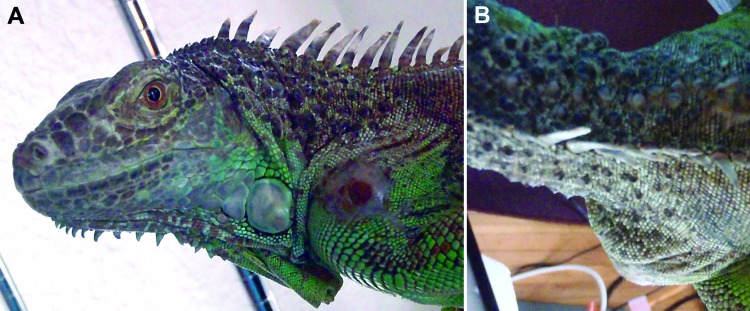
Abscess on shoulder of iguana infected with *Burkholderia pseudomallei*, California, USA, 2013. A) Lateral view. B) Dorsal view. Photo was taken at the time of sampling surface of animal and its environment.

Both owners stated that the iguanas had been purchased at young ages from local pet stores, had been kept indoors, and had not left California. The pathogen’s zoonotic potential was discussed with the owners and veterinarians in both cases. The owners were advised to consult with their physicians about potential exposure and were advised to use personal protective equipment (e.g., gloves, masks, and dedicated clothing) when handling the pets, to wash their hands afterward, and to disinfect the pets’ environment with 0.5%–1% sodium hypochlorite. No pet-related *B. pseudomallei* infections in humans have been reported.

## Conclusions

This report identifies *B. pseudomallei* isolates in 2 pet green iguanas and the environment of one of the infected pets. Their clinical disease developed when they were adults and was refractory to antimicrobial drug treatment. The second iguana suffered mild trauma shortly before its abscess appeared. Human case reports suggest that trauma may trigger the onset of disease (*14*).

Most green iguanas entering the pet trade in the United States originate from Central America (*15*). Both iguanas in this report and a person exposed in Costa Rica were infected with isolates with an identical sequence type by MLST. These results suggest that the iguanas were infected in Central America and experienced prolonged incubation periods (>1.5 years).

Positive PCR results from the water bowl suggest environmental contamination. The open abscess on the second iguana tested positive by both PCR and culture and was the likely source. However, negative PCR and culture results from the iguana’s vent, feet, and feces in the water bowl suggest contamination may not have been widespread.

Reptile owners, sellers, veterinarians, and staff in veterinary laboratories should be aware that green iguanas with abscesses may be infected with *B. pseudomallei* and that they should use appropriate personal protective equipment when handling them. Laboratories accepting samples from animals possibly infected with *B. pseudomallei* should anticipate risks associated with culturing this bacteria and take steps to protect workers.
